# Push-Out Bond Strength of EndoSeal Mineral Trioxide Aggregate and AH Plus Sealers after Using Three Different Irrigation Protocols

**DOI:** 10.1055/s-0041-1742128

**Published:** 2022-02-23

**Authors:** Shimaa Rifaat, Ahmed Rahoma, Fatimah Alkhalifa, Ghofran AlQuraini, Zahraa Alsalman, Zahraa Alwesaibi, Noha Taymour

**Affiliations:** 1 Department of Restorative Dental Sciences, College of Dentistry, Imam Abdulrahman Bin Faisal University, Dammam, Saudi Arabia; 2 College of Dentistry, Imam Abdulrahman Bin Faisal University, Dammam, Saudi Arabia; 3 Department of Substitutive Dental Sciences, College of Dentistry, Imam Abdulrahman Bin Faisal University, Dammam, Saudi Arabia

**Keywords:** EDTA, push-out, irrigating solution, bond strength, AH plus, maleic acid, EndoSeal MTA

## Abstract

**Objective** The current study was designed to assess the bonding strength of EndoSeal MTA and AH Plus sealers after using three irrigation protocols as follows: (1) 17% Ethylenediamine tetraacetic acid, (2) 7% maleic acid, and (3) 37% phosphoric acid.

**Materials and Methods** Push-out bond strength was evaluated for 60 middle root slices of 1-mm thickness each. They were horizontally cut from freshly extracted single-rooted human teeth. A hole in the root canal was made using a carbide round bur of 1.1 mm in diameter in a middle third root slice. Specimens were dipped in 2.5% NaOCl, and then they were grouped into three groups; G1: 17% EDTA, G2: 7% maleic acid, and G3: 37% phosphoric acid as a final irrigant for 3 minutes. Each group was subdivided into two subgroups, according to the type of sealer, either EndoSeal MTA or AH Plus.

**Statistical Analysis** After the full set of the sealer, the bond strength was evaluated with the push-out test by applying a force to each slice using a plunger with a 1-mm diameter. The one-way Tukey's post hoc test, analysis of variance (ANOVA) test, and Student's *t* -test were utilized to gather data and statistically evaluate it.

**Results** The irrigation protocol used exhibited significant influence on the bond strength of EndoSeal MTA and AH Plus sealers. AH Plus sealer subgroups showed the highest bond strength with 7% maleic acid, followed by 37% phosphoric acid, and 17% EDTA. While in the EndoSeal MTA sealer subgroups, the highest bond strength was shown with the 17% EDTA followed by 7% maleic acid and 37% phosphoric acid, respectively.

**Conclusion** The present study revealed that the type of the final irrigant significantly impacts the bond strength of the sealer used. The AH Plus sealer bond strength was improved by using the 7% maleic acid as a final irrigant. In contrast, the EndoSeal MTA sealer showed the best results with the 17% EDTA as a final irrigant.

## Introduction

Successful root canal treatment relies on the proper cleaning of the canal and complete removal of the microorganisms. Moreover, using an inert root canal filling material that can fill the canal space three dimensionally and inhibit any bacterial invasion from the oral cavity to the periapical tissues can improve root canal treatment success rates. 1

The most used irrigating solution in the endodontic field is sodium hypochlorite (2.5% NaOCl). It is widely used because of its capability to dissolve the organic tissues inside the canal. However, its inability to dissolve the inorganic materials is a drawback. The existence of the smear layer on the canal walls negatively affects the bond strength at the root sealer–dentine interface. 2 Many demineralizing agents have been used as adjunctive to the 2.5% NaOCl to ensure the hybridized smear layer elimination. 3

EDTA and sodium hypochlorite combination is usually used to eliminate the smear layer efficiently from the root canal system. 1 Smear layer removal time ranges from 1 to 10 minutes on using 17% EDTA. Nevertheless, it was reported that this combination might lead to erosion of the peritubular and intertubular dentin compared with distilled water and decrease the microhardness of dentin. 4 However, it was reported that 3-minute exposure of the root dentin to 17% EDTA showed complete removal of the smear layer. 5

Several conditioning materials were used for surface treatment for either enamel or dentin like phosphoric acid and maleic acid. Maleic acid is used as a mild organic acid conditioner in adhesive dentistry due to its smear layer removal ability. 5 Different studies have been conducted to evaluate the various possible concentrations of maleic acid for smear layer removal from root canals. Prabhu et al 6 stated that using more than 7% concentration of maleic acid may lead to erosion of the intertubular dentin. At the same time, other studies showed that 37% phosphoric acid concentration led to the complete elimination of the smear layer from the root dentin. 7

AH Plus resin sealer is the most used endodontic sealer due to its favorable physical and biological properties, apical sealability, low solubility, and adhesion to root dentin. 7 In 1995, Torabinejad and White 8 introduced the first bioceramic material in the endodontic field, the Mineral Trioxide Aggregate (MTA). It is a biocompatible, inductive, and osteoconductive material. 3 Moreover, the desirable MTA sealing ability is due to its calcium ions release and the production of an apatite layer with phosphates containing physiologic fluids. A pozzolan-based root canal sealer EndoSeal MTA has the original Mineral Trioxide Aggregate same physical and biological characteristics. 9

Different irrigation protocols have been used to achieve adequate bond strength between endodontic sealer and root dentin. Ulusoy et al found that the maleic acid has a remarkable decrease in the bond strength of resin sealers when compared with EDTA. 10 However, in a similar study, it was found that the Maleic acid showed significant improvement in the bond strength of resin sealers in comparison to EDTA. 11

Till present, no definitive research has been concluded the optimum bond strength for EndoSeal MTA and AH Plus sealers after using different irrigation protocols. Hence, the present research was designed to compare the efficacy of 17% EDTA, 7% maleic acid, and 37% phosphoric acid on both types of sealers using the push-out bond strength test.

In this work, the null hypothesis was that there was no difference in the impact of the studied irrigation procedures on the bond strength of the sealers utilized.

## Materials and Methods

### Sample Size Calculation

The sample size estimate was calculated using the sample size calculation formula provided by the World Health Organization according to earlier research. 12 The of power of study *β* = 80% and margin of error *α* = 5%, *μ* 1, and *μ* 2 were the mean level, standard deviation ( *σ* ). A total size of 60-disc samples was required for this study, 20 discs for each group.

*n* = ( *Z _α_*_/2_ + *Z _β_* ) ^2^ *2* *σ* ^2^ / ( *μ* 1– *μ* 2) ^2^

### Sample Selection

Ethical permission was acquired from the Health Ethics Committee of Imam Abdulrahman Bin Faisal University, Dammam, Saudi Arabia (EA: 2019019). A total of 60 single-rooted teeth, freshly extracted, at least 15-mm root length, and completely formed apices were used. Preoperative X-rays for each tooth were prepared for further examination. Teeth with internal resorption, calcification, cracks, curved, or narrow canals, or any structural defect had been excluded. They were stored in 10% formalin immediately after extraction. The 10% formalin is the only disinfectant material penetrating the pulp chamber and is considered an effective antimicrobial agent. 13 Moreover, Pichardo et al suggested that 10% formalin can stabilize the collagen inside the dentinal tubules and prevent their collapse, allowing more mechanical interlocking of the restorative material to the tubules and preventing microleakage. 13

### Sample Preparation

**Fig. 1:**
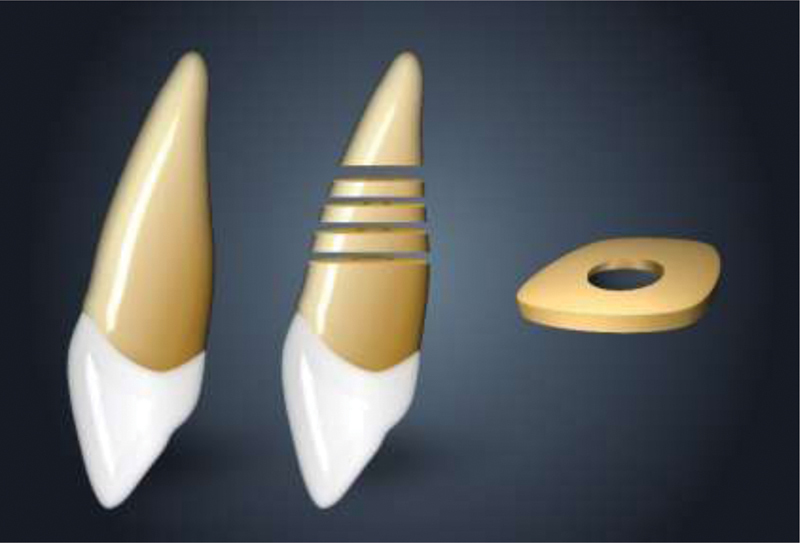
Schematic illustration of sample preparation.

Specimens were mounted in acrylic mold and decoronated to obtain a 15-mm root length in all samples utilizing a double-faced diamond disc (KG Sorensen, Ind. Com. Ltda.; Barueri, Sao Paolo, Brazil). A precision diamond saw (Isomet 1000, Lake Buff, Buehler, Illinois, United States) was used to cut 1-mm horizontal cross-sectional slices perpendicular to the long tooth axis from the middle root third along with a water coolant system Fig. 1 . One middle root third slice per tooth resulted in a total of 60 slices being collected for further investigation. A digital caliper was used to check the thickness of each slice (Pachymeter, Electronic Digital Instruments, China).

Every slice was assigned a code and calibrated through comparison to an item of an identified length, like a ruler in the present study.

A vertical drill was used to drill a standardized hole with a 1.1-mm cylindrical carbide bur (893–047, Brasseler, Savannah, Georgia, United States) and constant water irrigation. The drilled hole standardized the root canal anatomy and diameter among the specimens, increasing the push-out model's internal validity, and providing more control over the failure mode. Samples were dipped for 15 minutes in 2.5% NaOCl solution (Sodium Hypochlorite Lot 050613–9, Fair Lawn, New Jersey) followed by bidistilled water for 1 minute to neutralize sodium hypochlorite's effect. Samples were distributed in three groups with 20 slices each. Each group was irrigated with one of the following 17% EDTA (PulpDent, Watertown, Massachusetts, United States), 7% maleic acid (KMC Pharmacy, Manipal, Karnataka, India), or 37% phosphoric acid (Total Etch, Ivoclar Vivadent AG, Liechtenstein) as a final irrigant for 3 minutes as recommended in the literature. 4 14 The drilled holes were dried with paper points. After that, the three final irrigant groups were divided into two subgroups each. The two subgroups were filled with either EndoSeal MTA (EndoSeal, Maruchi, Seoul, Korea) or AH Plus sealers (Dentsply, DeTrey, Konstanz, Germany). Sealers were prepared as per the manufacturer's guidelines and inserted into the holes. Gentle vibration was performed while placing the sealer to avoid any bubble formation. The filled slices were kept in phosphate-buffered saline at 37°C (pH = 7.2) for 7 days before the assessment to ensure the sealer's full set.

### Assessment of the Push-Out Bond Strength

**Fig. 2:**
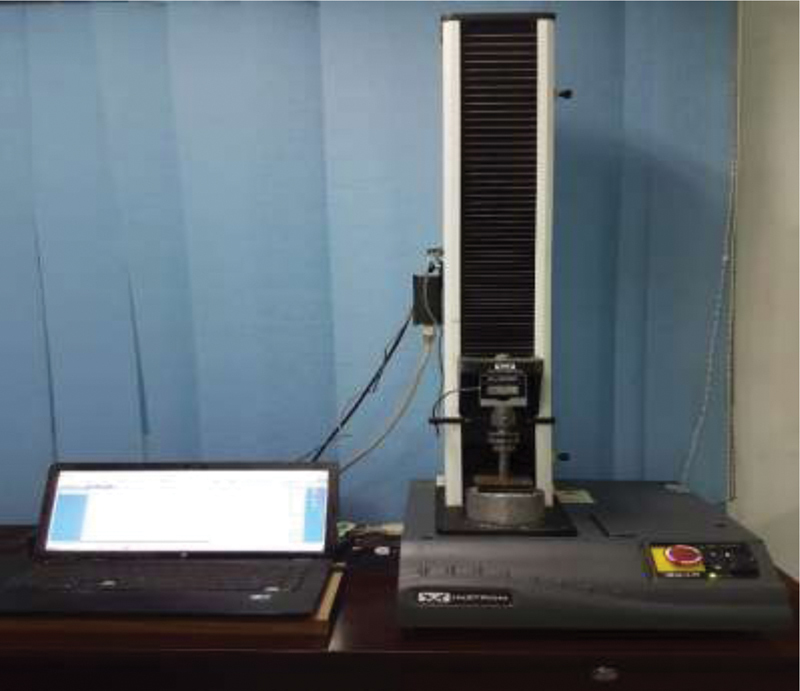
Push-out testing machine (Instron Industrial Products, Model 3345; Massachusetts, United States).

**Fig. 3:**
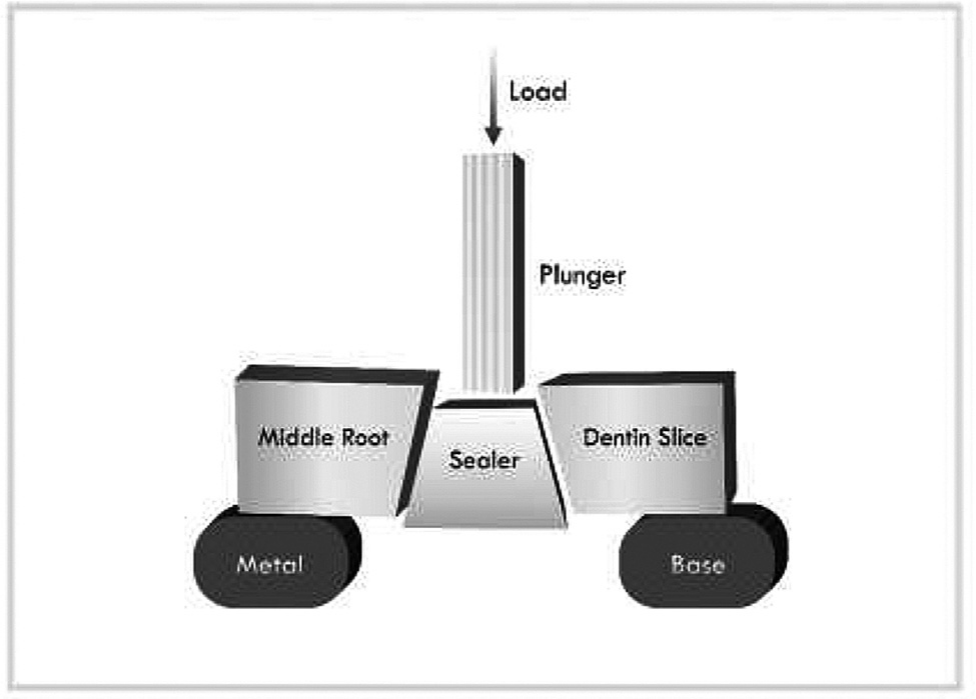
Push-out test design.

Through a computer-controlled testing machine, all the slices were exposed to compressive force at a 1 mm/min crosshead speed (Instron Industrial Products, Model 3345; Massachusetts, United States; Fig. 2 ). The load on the sealer that filled the slices was applied using a plunger with a 1-mm diameter equivalent to the root third to be tested. The plunger tip was placed such that it just touched the sealer filling and did not put any strain on the surrounding dentin. This positioning was accomplished in an apicocoronal direction to push the sealer filling toward the large diameter of the slice, thus preventing any filling movement limitation. Hence, the overlying dentin was supported adequately throughout the process of compressive stress Fig. 3 .

The maximum adhesive failure load was measured in Newton and then changed to MPa. Bond strength was divided and determined by the computed surface area using the formula of (A = 2 [3.14 × r × h], where; r is the radius, and h is the sample thickness in mm). Extrusion of the sealer piece was counted as an adhesive failure. The bond strength was concluded for each slice. 15 16

## Statistical Analysis

The Statistical Package for the Social Sciences (SPSS Co., Chicago, Illinois, United States) Software version was used to perform the statistical analysis. One-way analysis of variance (ANOVA) test, Tukey's post hoc test, and Student's *t* -test were utilized respectively with a significance level of *p* ≤ 0.05 to estimate the influence of the variables on the bond strength, where the irrigation solution and the sealers are the independent variables.

## Results

Data were collected, the standard deviation (SD) and mean values were calculated. A Graph Pad InStat software for Windows (GraphPad, Inc.) was utilized to analyze the results. A statistically significant value is when *p* ≤ 0.05. After variance homogeneity and standard errors distribution had been confirmed, Tukey's post hoc, one-way ANOVA, and Student's *t* -test were used to identify the significance among all the tested groups.

### Push-Out Bond Strength Test Results

SDs and means for the push-out bond strengths (in MPa) were collected for the tested root canal sealers and illustrated in Table 1 . A significant difference was recognized among various groups.

**Table 1:** Push-out bond strength results (mean values ± SDs) for both sealer groups after using three different irrigants

Variables		Sealer		*t* -Test
		MTA	AH Plus	*p* -Value
Final irrigant	EDTA	1.379 a ± 0.22	0.881 c ± 0.52	0.0356 d
	H _3_ PO _4_	0.494 b ± 0.13	1.915 b ± 0.19	<0.0001 d
	Maleic	1.317 a ± 0.27	2.377 a ± 0.19	<0.0001 d
ANOVA test	*p* -Value	<0.0001 d	<0.0001 d	

Abbreviations: ANOVA, analysis of variance; MTA, mineral trioxide aggregate; SD, standard deviation.

a,b,c Different letters in the same column indicating statistically significant difference ( *p* < 0.05).

d Significant ( *p* < 0.05) and nonsignificant ( *p* > 0.05).

Note: Push out bond strength test results: When comparing the results of the different final irrigants with the EndoSeal MTA and the AH Plus sealers (Vertically), the one-way ANOVA test was used. That had been followed with Tukey's post hoc test to analyze the similar final irrigation responses to the lowest one. Comparing the effect of 17% EDTA on EndoSeal MTA and AH Plus sealers (horizontally), the Student *t* -test was used ( *p* ≤ 0.05).

**Chart 1:**
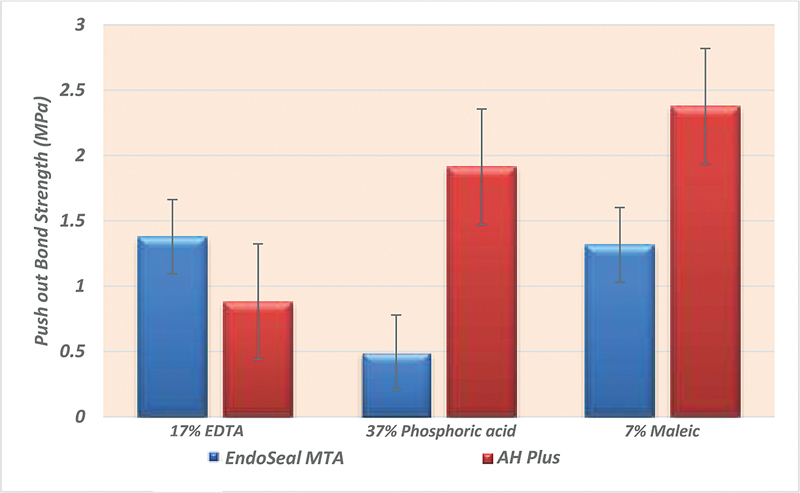
The mean values of push-out bond strength for both sealer groups with different surface treatments. MTA, mineral trioxide aggregate.

The current study revealed that the EndoSeal MTA sealer subgroups recorded significantly higher push-out bond strength value when 17% EDTA was used as a final irrigant, followed by 37% phosphoric acid groups. While the significantly low value of the push-out bond strength was detected with the 7% maleic acid group, as shown in Table 1 and Chart 1 .

Regarding the AH Plus sealer subgroups, the significantly higher push-out bond strength value was detected with the 7% maleic acid final irrigant, followed by the 37% phosphoric acid. However, the statistically significant low value was recorded with the 17% EDTA group, as illustrated in Table 1 and Chart 1 .

**Chart 2:**
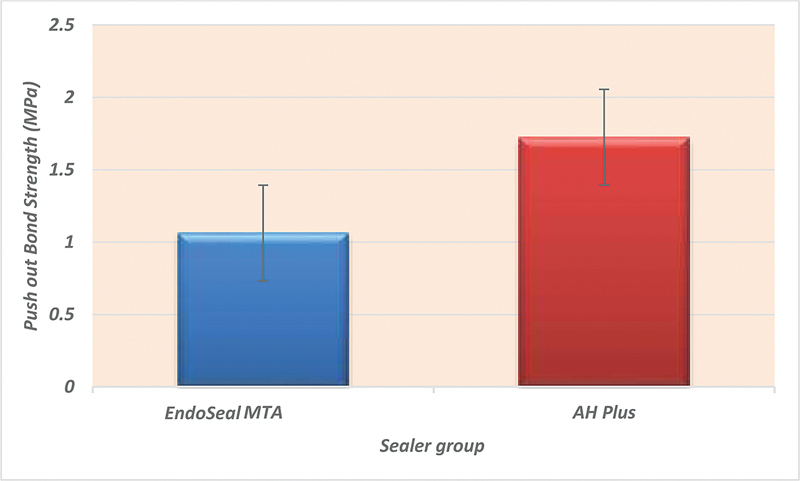
Comparison of the total mean values of push-out bond strength as function of sealer group. MTA, mineral trioxide aggregate.

Table 2 and Chart 2 showed a higher value for the AH Plus sealer in comparison to the EndoSeal MTA sealer, although no significant difference was detected between both sealer groups.

**Table 2:** Comparison of total push-out bond strength results (mean values ± SDs) between EndoSeal MTA and AH Plus sealer groups

Variables		Mean ± SDs	Rank	Statistics
Sealer group	MTA	1.063 ± 0.38	B	*p* -Value
	AH Plus	1.725 ± 0.56	A	0.3168

Abbreviations: MTA, mineral trioxide aggregate; SD, standard deviation.

Notes: Statistically significant ( *p* < 0.05) and nonsignificant ( *p* > 0.05).

Total effect of sealer group on push-out bond strength: Note: comparing the EndoSeal MTA and AH Plus sealers with all final irrigants, Student's *t* -test was used ( *p* ≤ 0.05).

## Discussion

Entrapment of a material into another body, either natural or artificial, is the mechanism through which mechanical adhesion originates. 17 Therefore, the bond strength of the endodontic sealers depends on the smear layer elimination to enhance sealer infiltration into the dentinal tubules.

The push-out bond strength test is commonly used to assess the dislodgement resistance of the root canal filling materials. 18 19 Several studies have shown that higher bond strengths could be recognized for the AH Plus sealers than others. 20 Furthermore, AH Plus is thought to be an ideal model against which other new sealers can be compared. 21 22 23 Consequently, in the current study, we targeted to assess the push-out bond strength of EndoSeal-MTA and AH Plus sealers and compare their values after using three different irrigation protocols.

Results presented that the higher bond strength was shown with the AH Plus sealer when used with 7% maleic acid and 37% phosphoric acid as a final irrigant compared with 17% EDTA. This might be related to the low surface tension of the 7% maleic acid (0.06345 N/m) and the 37% phosphoric acid (0.00746 N/m) when compared with 17% EDTA (0.0783 N/m). 24 25 Moreover, both 7% maleic and 37% phosphoric acids are highly acidic, thus having a superior demineralizing effect within a shorter duration. In comparison, the efficiency of 17% EDTA reduced over time due to the pH reduction. 26 27 This agrees with Ballal et al 28 they studied the results of using 7% maleic acid on the push-out bond strength of the AH Plus sealer. They concluded that the 7% maleic acid alone or in combination with cetrimide increased the root dentin wettability and thus raised the bond strength at the root dentin–sealer interface. 29

Furthermore, the bond strength of the AH Plus sealer to the root dentin rinsed with 7% maleic acid was significantly higher than that treated with 37% phosphoric acid as the final irrigant. The reason behind this result is that 7% of maleic acid produced the highest surface roughness compared with other irrigation solutions. 30

A significantly low push-out bond strength (0.881 ± 0.52 MPa) was observed when the EDTA was used as a final irrigant prior to using the AH Plus sealer. This was demonstrated by the 17% EDTA's low demineralizing ability and lack of a surfactant effect. As a result, a demineralized collagen fibril of a very thin layer was formed on the dentine surface. This layer is responsible for the weak wettability of the AH Plus root canal sealer on the 17% EDTA-irrigated dentine. 31 32 Results of the present study come in accordance with Ballal et al, 14 they stated that the contact angle of resin sealers is increased after irrigating with 17% EDTA compared with other tested irrigating materials. Hence, the resin-based sealers' wettability or spreading on root canal dentine was decreased when using 17% EDTA as the final irrigant. These findings are comparable with those stated in several earlier research. 33

On the other hand, an interesting finding in this study was that the bond strength of the EndoSeal MTA sealer revealed higher results with the usage of 17% EDTA as a final irrigant compared with 7% maleic and 37% phosphoric acids. This might be attributed to the MTA-based sealers showing greater flow than epoxy resin sealers. Thus, the smear layer fuses with the EndoSeal MTA sealer mass and adds volume to the sealer penetrating the dentinal tubules. 34 This outcome agrees with Kuchi et al. 35 They investigated the effect of the smear layer presence on the penetration of the Metapex fill sealers and concluded that the smear layer presence does not negatively affect the sealer infiltration into the dentinal tubules. This may be a possible explanation for the higher bond strength of the tested EndoSeal MTA sealer in the current study. 36

The push-out bond strength of the EndoSeal MTA sealer was significantly less for phosphoric acid than for 17% EDTA and 7% maleic acid, which could be explained by the demineralization ability of the phosphoric acid being time dependent. 37

The results showed greater bond strength for the AH Plus sealer than the EndoSeal MTA sealer when comparing both EndoSeal MTA and AH Plus sealers in terms of the push-out bond strength, as shown in Table 2 . However, the results were nonsignificant, which could be explained by the inherent high adhesion ability of the AH Plus sealer in comparison to other sealers. 25 Thus, the null hypothesis theory was totally rejected.

Several research studies investigated the relationship between the smear layer and the sealer infiltration into the dentinal tubules. However, a group of researchers has stated that the presence of the smear layer restricts the sealer infiltration. 38 39 At the same time, others demonstrated that the smear layer does not impede the sealer penetration. 40 Another in vivo research has stated that sealer penetration happens remarkably despite a thick smear layer. 41 Our outcomes for the EndoSeal MTA sealer partly agree with the second group of studies. 42

## Conclusion

Within the limitations of this study, the model utilized in the current research, does not simulate the clinical condition. Moreover, the sealer detachment may be noticed during root sectioning as reported by Gesi et al. 42 In addition, the failure of adhesion could be mainly affected by the interface sealer/core material, meanwhile in the current study, no core material was used. Furthermore, the anatomical variations from one root to another and among the same root should be considered. It was determined increasing the bond strength of root canal sealers could be achieved by selecting the proper irrigation protocol. It appears that using the EndoSeal MTA sealer is preferable after applying 17% EDTA as a final irrigant. In comparison, the AH Plus sealer bond strength is preferable after using 7% maleic acid as a final rinse. However, further clinical studies are needed to confirm these results and evaluate their significance to the treatment outcome.

## References

[1] Dotto L Sarkis Onofre R Bacchi A Rocha Pereira G K Effect of root canal irrigants on the mechanical properties of endodontically treated teeth: a scoping review J Endod 2020 46 05 596 604000 32147184 10.1016/j.joen.2020.01.017

[2] Akyuz Ekim S N Erdemir A Effect of different irrigant activation protocols on push-out bond strength Lasers Med Sci 2015 30 08 2143 2149 26022731 10.1007/s10103-015-1772-z

[3] Ertas H Kucukyilmaz E Ok E Uysal B Push-out bond strength of different mineral trioxide aggregates Eur J Dent 2014 8 03 348 352 25202215 10.4103/1305-7456.137646 PMC4144133

[4] Rath P P Yiu C KY Matinlinna J P Kishen A Neelakantan P The effect of root canal irrigants on dentin: a focused review Restor Dent Endod 2020 45 03 e39 32839720 10.5395/rde.2020.45.e39 PMC7431934

[5] Ballal N V Kandian S Mala K Bhat K S Acharya S Comparison of the efficacy of maleic acid and ethylenediaminetetraacetic acid in smear layer removal from instrumented human root canal: a scanning electron microscopic study J Endod 2009 35 11 1573 1576 19840650 10.1016/j.joen.2009.07.021

[6] Prabhu S G Rahim N Bhat K S Mathew J Comparison of removal of endodontic smear layer using sodium hypochlorite, EDTA and different concentrations of maleic acid – a SEM study Endodontology 2003 15 20 25

[7] Ibrahim I M Elkassas D W Yousry M M Effect of EDTA and phosphoric acid pretreatment on the bonding effectiveness of self-etch adhesives to ground enamel Eur J Dent 2010 4 04 418 428 20922162 PMC2948749

[8] Torabinejad M White D J inventor; Loma Linda University, Loma Linda, assigneeTooth filling material and method of use. US patent 5,769,638. June 23, 1998

[9] Donnermeyer D Bürklein S Dammaschke T Schäfer E Endodontic sealers based on calcium silicates: a systematic review Odontology 2019 107 04 421 436 30554288 10.1007/s10266-018-0400-3

[10] Ulusoy Öİ Nayir Y Celik K Yaman S D Apical microleakage of different root canal sealers after use of maleic acid and EDTA as final irrigants Braz Oral Res 2014 28 1 6 25229787

[11] Neelakantan P Subbarao C Subbarao C V De-Deus G Zehnder M The impact of root dentine conditioning on sealing ability and push-out bond strength of an epoxy resin root canal sealer Int Endod J 2011 44 06 491 498 21255047 10.1111/j.1365-2591.2010.01848.x

[12] Rahoma A AlShwaimi E Majeed A Push-out bond strength of different types of mineral trioxide aggregate in root dentin Int J Health Sci (Qassim) 2018 12 05 66 69 30202410 PMC6124834

[13] Pichardo M R George S W Bergeron B E Jeansonne B G Rutledge R Apical leakage of root-end placed SuperEBA, MTA, and Geristore restorations in human teeth previously stored in 10% formalin J Endod 2006 32 10 956 959 16982272 10.1016/j.joen.2006.07.011

[14] Ballal N V Tweeny A Khechen K Prabhu K N Tay F R Wettability of root canal sealers on intraradicular dentine treated with different irrigating solutions J Dent 2013 41 06 556 560 23603234 10.1016/j.jdent.2013.04.005

[15] Nagas E Uyanik M O Sahin C Durmaz V Cehreli Z C Effects of different light-curing units and obturation techniques on the seal of the Resilon/Epiphany system J Endod 2008 34 10 1230 1232 18793927 10.1016/j.joen.2008.07.011

[16] Silva E JNL Carvalho N K Prado M C Push-out bond strength of injectable pozzolan-based root canal sealer J Endod 2016 42 11 1656 1659 27641946 10.1016/j.joen.2016.08.009

[17] Erickson R L Surface interactions of dentin adhesive materials Oper Dent 1992 05 81 94 1470557

[18] Neelakantan P Ahmed H MA Wong M CM Matinlinna J P Cheung G SP Effect of root canal irrigation protocols on the dislocation resistance of mineral trioxide aggregate-based materials: a systematic review of laboratory studies Int Endod J 2018 51 08 847 861 29377170 10.1111/iej.12898

[19] Ok E Ertas H Saygili G Gok T Effect of photo-activated disinfection on bond strength of three different root canal sealers Eur J Dent 2014 8 01 85 89 24966752 10.4103/1305-7456.126252 PMC4054038

[20] Tagger M Tagger E Tjan A H Bakland L K Measurement of adhesion of endodontic sealers to dentin J Endod 2002 28 05 351 354 12026917 10.1097/00004770-200205000-00001

[21] Sönmez I S Sönmez D Almaz M E Evaluation of push-out bond strength of a new MTA-based sealer Eur Arch Paediatr Dent 2013 14 03 161 166 23645545 10.1007/s40368-013-0039-2

[22] Brackett M G Martin R Sword J Comparison of seal after obturation techniques using a polydimethylsiloxane-based root canal sealer J Endod 2006 32 12 1188 1190 17174680 10.1016/j.joen.2006.07.009

[23] Jainaen A Palamara J E Messer H H Push-out bond strengths of the dentine-sealer interface with and without a main cone Int Endod J 2007 40 11 882 890 17877721 10.1111/j.1365-2591.2007.01308.x

[24] Jagzap J B Patil S S Gade V J Chandhok D J Upagade M A Thakur D A Effectiveness of three different irrigants-17% ethylenediaminetetraacetic acid, Q-MIX, and phytic acid in smear layer removal: a comparative scanning electron microscope study Contemp Clin Dent 2017 8 03 459 463 29042735 10.4103/ccd.ccd_524_17 PMC5644007

[25] Paqué F Luder H U Sener B Zehnder M Tubular sclerosis rather than the smear layer impedes dye penetration into the dentine of endodontically instrumented root canals Int Endod J 2006 39 01 18 25 16409324 10.1111/j.1365-2591.2005.01042.x

[26] Ballal N V Jain I Tay F R Evaluation of the smear layer removal and decalcification effect of QMix, maleic acid and EDTA on root canal dentine J Dent 2016 51 62 68 27287285 10.1016/j.jdent.2016.06.001

[27] Mohammadi Z Shalavi S Yaripour S Smear layer removing ability of root canal irrigation solutions: a review J Contemp Dent Pract 2019 20 03 395 402 31204334

[28] Ballal N V Ferrer-Luque C M Sona M Prabhu K N Arias-Moliz T Baca P Evaluation of final irrigation regimens with maleic acid for smear layer removal and wettability of root canal sealer Acta Odontol Scand 2018 76 03 199 203 29126370 10.1080/00016357.2017.1402208

[29] Kuruvilla A Jaganath B M Krishnegowda S C Ramachandra P KM Johns D A Abraham A A comparative evaluation of smear layer removal by using EDTA, etidronic acid, and maleic acid as root canal irrigants: an in vitro scanning electron microscopic study J Conserv Dent 2015 18 03 247 251 26069414 10.4103/0972-0707.157266 PMC4450534

[30] Mohammadi Z Jafarzadeh H Shalavi S Kinoshita J I Unusual root canal irrigation solutions J Contemp Dent Pract 2017 18 05 415 420 28512283 10.5005/jp-journals-10024-2057

[31] Plotino G Buono L Grande N M Pameijer C H Somma F Nonvital tooth bleaching: a review of the literature and clinical procedures J Endod 2008 34 04 394 407 18358884 10.1016/j.joen.2007.12.020

[32] Saleh I M Ruyter I E Haapasalo M Ørstavik D The effects of dentine pretreatment on the adhesion of root-canal sealers Int Endod J 2002 35 10 859 866 12406381 10.1046/j.1365-2591.2002.00585.x

[33] Dogan Buzoglu H Calt S Gümüsderelioglu M Evaluation of the surface free energy on root canal dentine walls treated with chelating agents and NaOCl Int Endod J 2007 40 01 18 24 17209828 10.1111/j.1365-2591.2006.01169.x

[34] Attal J-P Asmussen E Degrange M Effects of surface treatment on the free surface energy of dentin Dent Mater 1994 10 04 259 264 7664994 10.1016/0109-5641(94)90071-x

[35] Kuçi A Alaçam T Yavaş O Ergul-Ulger Z Kayaoglu G Sealer penetration into dentinal tubules in the presence or absence of smear layer: a confocal laser scanning microscopic study J Endod 2014 40 10 1627 1631 25260735 10.1016/j.joen.2014.03.019

[36] Valencia Y M Vertuan G C Alcalde M P Vivan R R Reis Só M V Duarte M AH Effect of irrigating agitation after root end preparation on the wall cleaning and bond strength of calcium silicate material in retrograde obturation Eur J Dent 2021 15 04 707 713 34303319 10.1055/s-0041-1729454 PMC8630967

[37] Sofan E Sofan A Palaia G Tenore G Romeo U Migliau G Classification review of dental adhesive systems: from the IV generation to the universal type Ann Stomatol (Roma) 2017 8 01 1 17 28736601 10.11138/ads/2017.8.1.001 PMC5507161

[38] Kokkas A B Boutsioukis ACh Vassiliadis L P Stavrianos C K The influence of the smear layer on dentinal tubule penetration depth by three different root canal sealers: an in vitro study J Endod 2004 30 02 100 102 14977306 10.1097/00004770-200402000-00009

[39] Okşan T Aktener B O Şen B H Tezel H The penetration of root canal sealers into dentinal tubules. A scanning electron microscopic study Int Endod J 1993 26 05 301 305 8300262 10.1111/j.1365-2591.1993.tb00575.x

[40] Kara Tuncer A Tuncer S Effect of different final irrigation solutions on dentinal tubule penetration depth and percentage of root canal sealer J Endod 2012 38 06 860 863 22595128 10.1016/j.joen.2012.03.008

[41] Vassiliadis L P Sklavounos S A Stavrianos C K Depth of penetration and appearance of Grossman sealer in the dentinal tubules: an in vivo study J Endod 1994 20 08 373 376 7996102 10.1016/S0099-2399(06)80293-1

[42] Gesi A Raffaelli O Goracci C Pashley D H Tay F R Ferrari M Interfacial strength of Resilon and gutta-percha to intraradicular dentin J Endod 2005 31 11 809 813 16249724 10.1097/01.don.0000158230.15853.b7

